# Whole lung directed anti-muscarinic therapy improves small airway dysfunction in COPD patients

**DOI:** 10.1183/13993003.01326-2025

**Published:** 2026-02-05

**Authors:** Omar S. Usmani, Dimitrios Toumpanakis, Sally Meah, Vincent Mak, Martyn F. Biddiscombe

**Affiliations:** 1 National Heart and Lung Institute, Imperial College London, London, UK; 2 2nd Department of Critical Care, “Attikon” University Hospital, National and Kapodistrian University of Athens, Athens, Greece; 3 Charing Cross Hospital, Imperial College Healthcare Trust, London, UK

## Abstract

Small airway disease is a key feature of COPD that often precedes emphysema, with the small airways representing a major site of airflow limitation [1]. The prevalence of small airway dysfunction (SAD) ranges from 50% to 90% in COPD patients, and correlates with symptoms [2]. SAD is usually well-established before conventional spirometry is abnormal [3], and contributes to patient quality-of-life burden [4] and disease exacerbations [5].

## Shareable abstract

**Directed distribution of inhaled therapy to diseased lung regions improves patient lung function. Small airway disease should be considered a treatable trait and actively sought in the clinic, leading to targeting therapeutic treatment to this region.** https://bit.ly/4oHAwGM

*To the Editor:*

Small airway disease is a key feature of COPD that often precedes emphysema, with the small airways representing a major site of airflow limitation [1]. The prevalence of small airway dysfunction (SAD) ranges from 50% to 90% in COPD patients, and correlates with symptoms [2]. SAD is usually well-established before conventional spirometry is abnormal [3], and contributes to patient quality-of-life burden [4] and disease exacerbations [5].

Inhalation therapy is the bedrock of COPD management, yet despite increasingly new inhaler device–drug combinations, COPD remains sub-optimally controlled for many patients. Most prescribed inhalers, in practice, deliver drugs primarily to the large airways [6]. The inability to effectively treat both the large airways and SAD may be a limiting factor in achieving optimal patient benefit [6]. Technological advances in inhaler engineering now allow targeting of the small airways [6], and coupled with contemporary progress in more sensitive physiological assessments for SAD [1], careful investigation of whether therapeutic targeting of small airways leads to improved patient lung function is now possible [5].

We hypothesised that inhaled tiotropium, a long-acting muscarinic antagonist, the mainstay of COPD pharmacological treatment, aerosolised to a high fine-particle fraction (FPF; ∼65–80%, Respimat soft-mist inhaler (RM-SMI)) [7], achieving both large airway deposition and deeper lung penetration to the small airways [8], would improve SAD better than tiotropium at a low FPF (∼19%, Handihaler dry-powder inhaler (HH-DPI)), which would predominantly target the proximal central airways [9].

45 patients were recruited; two withdrew after study entry, and 43 (20 females, mean age 69 years, range 46–87 years) symptomatic (COPD Assessment Test (CAT) score >10) patients (Global Initiative for Chronic Obstructive Lung Disease stage I–III, mean±sd forced expiratory volume in 1 s (FEV_1_) 65.8±17.0% of predicted) using HH-DPI (tiotropium 18 µg daily) for >6 months, and all naïve to RM-SMI, completed all visits. In this open-label sequential treatment, pragmatic real-life study, patients initially received on visit 1 (day 0) HH-DPI (tiotropium 18 μg daily, 14 days), then on visit 2 (day 14) were switched to RM-SMI (tiotropium 5 µg daily, 14 days). We did not undertake drug deposition imaging in this study.

At each visit, patients underwent, in order: pre-dose (trough, 24 h after last tiotropium dose) lung physiology assessment (multiple-breath nitrogen washout (MBNW), impulse oscillometry, body plethysmography and spirometry), symptom questionnaires (CAT, modified Medical Research Council (mMRC) dyspnoea scale), inhaler training by an investigator using placebo devices, investigator-observed first-dose active drug inhalation, followed by a 3-h post-dose lung physiology assessment. At closeout visit (day 28), after pre-dose lung physiology assessment, patients expressed their opinions about each inhaler. All patients had stable COPD with no previous (past 2 months) chest infection requiring antibiotics and/or oral corticosteroids. None had received triple therapy, nor a previous diagnosis of asthma. Active drug and placebo training inhalers were supplied by Boehringer Ingelheim, Germany. The study protocol was approved by ethics committee (REC number: 15/LO/0982) and registered at Clinical trials.gov (identifier: NCT02683668).

The primary end-point was oscillometry-determined R5–R20 (difference between resistance at 5 Hz and at 20 Hz), a measure of SAD [1]. The Welch's unequal variances t-test was used to compare trough lung function measures at day 28 (pre-dose) to day 14 (pre-dose), and day 14 (pre-dose) compared to day 0 (pre-dose) (GraphPad Prism); p<0.05 defined statistical significance.

All subjects had evidence of SAD, with abnormal MBNW-S_acin_ (male >0.083±0.030 L^−1^, female >0.099±0.032 L^−1^) [10], where S_acin_ (ventilation heterogeneity in the acinar lung zone) has been correlated with loss of terminal bronchioles [11]. Trough values of oscillometry parameters reflecting total (R5) and small (R5–R20) airway mechanics significantly improved following RM-SMI treatment, whereas no improvement was noticed for HH-DPI (table 1). At day 0, small and total airway oscillometry indices initially improved post-dose with HH-DPI compared to pre-dose trough values; however, after 14 days, the physiological improvement observed declined almost to pre-dose trough values. Similarly, treatment with RM-SMI improved small and total airway oscillometry indices 3 h post-dose but, in contrast to HH-DPI, there was continued improvement with increased physiological values on day 28, suggesting sufficient and consistent pharmacological dosing of tiotropium by RM-SMI to cause a sustained effect in the lung periphery.

**TABLE 1:** Demographics (age, sex assigned at birth, height and weight), lung function, and health status at days 0, 14 and 28

	Day 0		Day 14		Day 28
	Pre-dose	Post-dose	Pre-dose	Post-dose	Pre-dose
**Patient demographics**					
Patients (n)	43				
Age (years)	69.14 (46–87)				
Male (n)	23				
BMI (kg·m^−2^)	28 (20.0–39.9)				
Smoking (pack-years)	44.2 (0–120)				
Exacerbations in past year (n)	0.6 (0–2)				
mMRC dyspnoea score	2.4 (1–4)		2.3 (1–4)		2.3 (1–4)
CAT score	17.4 (4–31)		16.7 (4–34)		16.7 (0–29)
**Impulse oscillometry**					
R5 (kPa·s·L^−1^)	0.61 (0.26–1.06)	0.53 (0.28–1.11)	0.58 (0.29–1.10)	0.51 (0.25–0.94)	0.50 (0.27–1.02)*
R20 (kPa·s·L^−1^)	0.37 (0.20–0.69)	0.35 (0.21–0.61)	0.37 (0.21–0.63)	0.34 (0.19–0.55)	0.34 (0.2–0.60)
R5–R20 (kPa·s·L^−1^)	0.24 (0.04–0.56)	0.18 (0.03–0.52)	0.21 (0.03–0.62)	0.217(0.02–0.44)	0.16 (0.03–0.42)*
X5 (kPa·s·L^−1^)	−0.31 (−0.06– −0.76)	−0.25 (−0.03– −0.87)	−0.27 (−0.07– −0.92)	−0.24 (−0.08– −0.72)	−0.23 (−0.06– −0.62)
AX (kPa·L^−1^)	2.80 (0.20–7.70)	2.02 (0.17–7.58)	2.30 (0.16–9.80)	1.90 (0.32–6.07)	1.66 (0.19–6.40)
**Multiple breath nitrogen washout**					
S_cond_ (L^−1^)	0.05 (0.00–0.17)	0.04 (0.00–0.15)	0.04 (0.00–0.15)	0.04 (0.00–0.13)	0.04 (0.00–0.13)
S_acin_ (L^−1^)	0.33 (0.11–0.53)	0.33 (0.11–0.54)	0.34 (0.06–0.57)	0.33 (0.08–0.60)	0.35 (0.03–0.65)
FRC (L)	4.38 (1.71–7.42)	4.08 (1.68–6.89)	4.24 (1.61–8.05)	4.25 (1.43–8.09)	4.42 (1.51–9.83)
LCI2.5%	9.96 (1.59–18.00)	10.37 (1.75–17.19)	10.56 (4.91–16.35)	10.87 (6.87–16.24)	11.27 (3.54–17.59)
LCI5%	7.11 (1.59–9.60)	7.17 (1.75–10.50)	7.31 (6.03–9.96)	7.24 (5.47–10.80)	7.48 (3.54–10.79)
**Body plethysmography**					
RV (L)	3.62 (1.39–6.40)	3.63 (2.12–6.69)	3.58 (1.89–8.44)	3.36 (1.74–7.65)	3.44 (1.20–7.99)
VC (L)	3.23 (2.01–5.81)	3.32 (2.03–5.64)	3.37 (2.04–5.70)	3.39 (1.89–5.82)	3.38 (2.00–5.90)
TLC (L)	6.85 (4.49–10.54)	6.95 (4.29–9.90)	6.95 (4.41–11.14)	6.74 (4.20–11.70)	6.82 (4.40–12.07)
FRC (L)	4.64 (2.34–7.83)	4.61 (2.37–7.97)	4.63 (2.40–10.11)	4.40 (2.07–9.19)	4.45 (2.31–8.64)
*D*_LCO_ SB (mmol·min^−1^·kPa^−1^)	4.30 (1.90–7.52)	4.46 (1.88–7.10)	4.43 (2.24–7.81)	4.49 (2.03–7.88)	4.42 (2.50–7.85)
**Spirometry**					
FEV_1_ (L)	1.57 (0.72–2.67)	1.67 (0.75–2.82)	1.58 (0.51–2.56)	1.65 (0.77–2.80)	1.65 (0.74–2.65)
FEV_1_ (% predicted)	65.8 (33.2–98.1)	69.2 (38.6–100.8)	66.0 (30.6–112.0)	68.6 (39.9–104.3)	69.3 (35.1–116.5)
FVC (L)	2.92 (1.65–5.21)	3.09 (1.87–5.57)	3.03 (1.50–5.40)	3.13 (1.85–5.61)	3.07 (1.83–5.31)
FVC (% predicted)	97.1 (43.5–138.1)	101.6 (56.3–148.3)	100.9 (53.3–142.9)	104.3 (55.9–141.0)	102.8 (47.4–138.8)
FEV_1_/FVC	0.55 (0.34–0.74)	0.55 (0.36–0.74)	0.53 (0.34–0.75)	0.53 (0.32–0.75)	0.54 (0.37–0.78)
PEF (L·s^−1^)	4.54 (1.91–10.17)	4.64 (1.39–7.89)	4.43 (1.24–8.23)	4.94 (1.52–8.15)	4.79 (1.89–8.16)
PIF (L·s^−1^)	3.12 (1.00–5.74)	3.27 (1.11–6.14)	3.25 (1.02–7.21)	3.62 (0.98–7.94)	3.40 (1.22–6.40)
MEF_75_ (L·s^−1^)	2.17 (0.59–4.71)	2.37 (0.63–6.22)	2.14 (0.35–5.86)	2.26 (0.62–5.46)	2.26 (0.63–5.69)
MEF_50_ (L·s^−1^)	0.86 (0.25–2.02)	0.92 (0.27–2.27)	0.86 (0.14–2.48)	0.89 (0.23–2.36)	0.91 (0.28–3.37)
MEF_25_ (L·s^−1^)	0.22 (0.10–0.49)	0.25 (0.11–0.81)	0.22 (0.11–0.53)	0.22 (0.11–0.56)	0.23 (0.10–0.64)
MEF_25–75_ (L·s^−1^)	0.59 (0.19–1.24)	0.63 (0.22–1.65)	0.58 (0.14–1.68)	0.59 (0.16–1.39)	0.62 (0.20–2.05)

Data are presented as mean (range). Statistical comparisons *via* Welch's t-test. *: p≤0.05 (between pre-dose day 28 and pre-dose day 14). BMI: body mass index; mMRC: modified Medical Research Council; CAT: COPD Assessment Test; R5 and R20: respiratory resistances at frequencies of 5 Hz and 20 Hz; R5–R20: difference between resistance at 5 Hz and resistance at 20 Hz; X5: reactance at 5 Hz; AX: reactance area under the curve; S_cond_: ventilation heterogeneity in conducting airways; S_acin_: ventilation heterogeneity in the acinar lung zone; FRC: functional residual capacity; LCI2.5% and LCI5%: lung clearance index calculated at 2.5% and 5% of the starting nitrogen concentration; RV: residual volume; VC: vital capacity; TLC: total lung capacity; *D*_LCO_ SB: diffusion capacity of the lung for carbon monoxide, single breath; FEV_1_: forced expiratory volume in 1 s; FVC: forced vital capacity; PEF: peak expiratory flow; PIF: peak inspiratory flow; MEF_75_, MEF_50_, MEF_25_ and MEF_25–75_: maximum expiratory flow at 75%, 50%, 25% and 25–75% of FVC.

Despite the improvement in oscillometry indices, high-FPF treatment did not affect large airway (bronchodilatory) spirometry measures (FEV_1_ and peak expiratory flow) or conventional small airway spirometry markers (maximum expiratory flow at 25–75% of forced vital capacity), reflecting the known insensitivity of spirometry to detect SAD. There was no significant change in MBNW (ventilation heterogeneity in conducting airways (S_cond_), S_acin_, lung clearance index) or body plethysmography measures (residual volume, functional residual capacity, total lung capacity, diffusion capacity of the lung for carbon monoxide) for either treatment. No change in CAT or mMRC dyspnoea scores were observed. No adverse events were noted.

We show that deep lung delivery of high-FPF aerosolised tiotropium to symptomatic (CAT score >10) COPD patients with SAD attained significantly greater therapeutic responses in small (R5–R20) and total (R5) airway physiology measures compared to low-FPF tiotropium. Our study is a unique first, investigating regional airway responses to two different inhaler devices with different aerosolised lung deposition characteristics, using the same drug at bioequivalent doses.

Interestingly, we observed a disconnect between patients' perception of benefit and the measured clinical response, highlighting the behavioural complexities of the patient–inhaler–therapeutic interaction. Of the 13 (31% of the 43 total patients) who did not prefer RM-SMI, eight (62%) showed on average ∼30% physiological improvement in R5–R20. One patient had lung function deterioration despite preferring RM-SMI, and one patient who felt worse with RM-SMI had objectively improved function. Unsurprisingly, patients with greater inhaler errors at baseline (pre-dose) inhaler technique assessment experienced a greater decrease in lung function. In contrast, a subset of patients using HH-DPI well did not maintain lung function benefits in real-world administration. Patients using both inhalers exhibited errors related to device preparation, the inhalation manoeuvre, and breath-hold, despite their familiarity with the HH-DPI. All devices were checked at end of treatment period and none were faulty.

Despite >6 months HH-DPI use, 88% of subjects at baseline were not using their device correctly, with a variety of inhaler errors observed. Predictably, training in HH-DPI use improved 3 h post-dose treatment effects. We did not re-assess technique after each treatment period, given that technique is known to worsen over time. In contrast, all patients were naïve to RM-SMI and so took longer to train (15.5±2.5 min *versus* 7.5±2.2 min HH-DPI), yet achieved better physiological responses, suggesting the device–drug technology of RM-SMI is forgiving of mistakes in inhaler technique in everyday use. We switched treatments sequentially, from DPI-HH to RM-SMI without washout, simulating everyday clinical prescribing. We mitigated treatment order effects by thoroughly training patients to achieve optimal delivery from both inhalers. Indeed, our protocol favoured HH-DPI, with patients using it for >6 months but none experiencing prior use of RM-SMI, yet RM-SMI achieved additional therapeutic improvement over DPI-HH.

We reflect on some limitations. Peak inspiratory flow (PIF) was not objectively measured, where sub-optimal flows may have compromised HH-DPI responses [12]. Although adequate PIF values were achieved to operate HH-DPI in the clinic, this may not have been the case at home. Additionally, hyperinflated patients may have lacked adequate inhaled volume and inspiratory effort to effectively aerosolise and disperse the HH-DPI drug to the lungs [12]. To mitigate this, we comprehensively trained patients in inhaler technique, all gently exhaling to end-tidal volume before inhalation. Certainly, differences in inhaler adherence between treatments may have confounded effects, as diary card dosing entries were self-reported. Our study was not powered to assess patient outcomes or quality of life. The clinically important differences for oscillometry indices in COPD need to be established, as was recently undertaken in asthma [13].

In summary, our study shows that it is not only about which inhaled drug is given, but also where it reaches in the lungs, that matters. By virtue of the deposition characteristics and behaviour of aerosolised drugs, a targeted and effective distribution to the appropriate, diseased region of the lung, is hereby shown to achieve better physiological airway responses, which are not simply a result of correcting poor patient inhalation technique. SAD should be considered a phenotype and treatable trait [5], as it has direct relevance to COPD patient outcomes [6], and should be actively sought in the clinic with consideration given to targeted therapeutic treatment to this region.
